# King-Devick Test Performance and Cognitive Dysfunction after Concussion: A Pilot Eye Movement Study

**DOI:** 10.3390/brainsci11121571

**Published:** 2021-11-27

**Authors:** Doria M. Gold, John-Ross Rizzo, Yuen Shan Christine Lee, Amanda Childs, Todd E. Hudson, John Martone, Yuka K. Matsuzawa, Felicia Fraser, Joseph H. Ricker, Weiwei Dai, Ivan Selesnick, Laura J. Balcer, Steven L. Galetta, Janet C. Rucker

**Affiliations:** 1 Department of Neurology, New York University Grossman School of Medicine, New York, NY 10016, USA; doria.gold@nyulangone.org (D.M.G.); johnrossrizzo@gmail.com (J.-R.R.); hudson.te@gmail.com (T.E.H.); jnm2154@columbia.edu (J.M.); wd471@nyu.edu (W.D.); laura.balcer@nyulangone.org (L.J.B.); steven.galetta@nyulangone.org (S.L.G.); 2 Department of Physical Medicine & Rehabilitation, New York University Grossman School of Medicine, New York, NY 10016, USA; yuenshan.lee@nyulangone.org (Y.S.C.L.); amanda.childs@nyulangone.org (A.C.); yuka.matsuzawa@nyulangone.org (Y.K.M.); joseph.ricker@nyulangone.org (J.H.R.); 3 Department of Mechanical & Aerospace Engineering, New York University Tandon School of Engineering, New York, NY 11201, USA; 4 Department of Biomedical Engineering, New York University Tandon School of Engineering, New York, NY 11201, USA; 5 Department of Physical Medicine & Rehabilitation, MetroHeath System, Cleveland, OH 44109, USA; ffraser@metrohealth.org; 6 Department of Electrical & Computer Engineering, New York University Tandon School of Engineering, New York, NY 11201, USA; selesi@nyu.edu; 7 Department of Population Health, New York University Grossman School of Medicine, New York, NY 10016, USA; 8 Department of Ophthalmology, New York University Grossman School of Medicine, New York, NY 10016, USA

**Keywords:** concussion, King-Devick, rapid automatized naming tasks, saccades, inter-saccadic interval, cognitive dysfunction

## Abstract

(1) Background: The King-Devick (KD) rapid number naming test is sensitive for concussion diagnosis, with increased test time from baseline as the outcome measure. Eye tracking during KD performance in concussed individuals shows an association between inter-saccadic interval (ISI) (the time between saccades) prolongation and prolonged testing time. This pilot study retrospectively assesses the relation between ISI prolongation during KD testing and cognitive performance in persistently-symptomatic individuals post-concussion. (2) Results: Fourteen participants (median age 34 years; 6 women) with prior neuropsychological assessment and KD testing with eye tracking were included. KD test times (72.6 ± 20.7 s) and median ISI (379.1 ± 199.1 msec) were prolonged compared to published normative values. Greater ISI prolongation was associated with lower scores for processing speed (WAIS-IV Coding, *r* = 0.72, *p* = 0.0017), attention/working memory (Trails Making A, *r* = −0.65, *p* = 0.006) (Digit Span Forward, *r* = 0.57, *p* = −0.017) (Digit Span Backward, *r*= −0.55, *p* = 0.021) (Digit Span Total, *r* = −0.74, *p* = 0.001), and executive function (Stroop Color Word Interference, *r* = −0.8, *p* = 0.0003). (3) Conclusions: This pilot study provides preliminary evidence suggesting that cognitive dysfunction may be associated with prolonged ISI and KD test times in concussion.

## 1. Introduction

A concussion is a form of mild traumatic brain injury in which biomechanical forces to the head or body result in neurological symptoms such as headaches, dizziness, blurred vision, emotional lability, difficulty concentrating, or slowed information processing [1]. In most individuals, symptoms resolve spontaneously within days to weeks following injury. However, prolonged recovery with persistent symptoms occurs in 10–25% of individuals [2,3,4]. Given the lack of symptom specificity and clarity with regard to the timing of biological concussion resolution, protracted recovery with persistent symptoms may overlap with the development of other disorders such as depression and psychosocial maladjustment. Although helpful, self-reporting of subjective symptoms of concussion cannot be completely relied upon for diagnosis, as athletes have been shown to under-report or even deny symptoms in order to return to play [5,6,7,8,9]. As a result, sensitive sideline and outpatient diagnostic tests are needed. 

Sideline diagnostic tests [10] include the Sports Concussion Assessment Tool (SCAT) Symptom Checklist [11,12], Standardized Assessment of Concussion (SAC) [13], Balance Error Scoring System (BESS) [14], Vestibular–Ocular Motor Screening (VOMS) test [15], and rapid automatized naming tests such as the King-Devick (KD) test of number-naming [16] and the Mobile Universal Lexicon Evaluation System (MULES) test of picture-naming [17]. These diagnostic tests are also increasingly utilized in the outpatient setting where concussions often arise from non-sports-related injuries and where long-lasting symptoms are common [18,19]. It is important to emphasize that the diagnosis of concussion remains a clinical diagnosis and cannot be entirely confirmed or refuted with any single diagnostic test. 

Completion of the KD test requires reading numbers with variable spacing on three test cards as rapidly as possible. Scores are generated based on the total time taken to complete the test. The KD test is a performance measure that involves attention, number recognition, language retrieval, and saccadic eye movements. Worsening of the time to complete the test relative to a baseline time is consistent with the diagnosis of concussion, since test scores normally improve with practice and are not affected by physical activity [20,21,22,23,24,25]. In the outpatient setting, the KD score obtained during the initial visit for concussion evaluation has been shown to predict the total number of visits and the total number of referrals (e.g., to neuropsychology, vestibular, or vision therapy). A greater number of visits and referrals are indicators of more severe or prolonged symptoms [19]. 

In an effort to understand behaviors associated with slowed KD test times in concussion, quantitative analysis of eye movements (i.e., eye tracking) previously revealed that prolonged KD times in a concussion cohort with protracted post-concussive symptoms were associated with greater numbers of saccades, larger deviations of saccadic endpoints (dysmetria), and a prolongation of the inter-saccadic interval (ISI), as compared to healthy controls [26]. ISI, defined as the time between saccades, was strongly correlated with KD times. This complex interval of time captures several important aspects of test performance, including duration of fixation time on the current number, name retrieval and verbalization of the current number, attentional disengagement from the current number, planning of the saccade to the next number, and saccadic latency, as well as concentration and neurobehavioral contributions. Although these components of the ISI are intermingled and unable to be realistically compartmentalized, this pilot study aimed to retrospectively examine the relation between ISI prolongation on the KD test and cognitive performance on standard neuropsychological assessments in individuals with a history of concussion and persistent symptoms. The hypothesis of the study was that KD performance and ISI duration would relate to attention and processing speed.

## 2. Materials and Methods

### 2.1. Participants

Data from participants with a history of concussion and self-reported persistent symptoms who had previously completed a standardized clinical neuropsychological assessment and KD testing with eye tracking as part of a research protocol were retrospectively reviewed. All participants provided written informed consent to participate in eye tracking research and in a concussion registry database that included neuropsychological assessment data. The study was approved by the New York University Grossman School of Medicine Institutional Review Board (S13-01229 and S14-02097). Exclusion criteria included incomplete neuropsychological assessment, visual impairment precluding KD performance, and moderate-severe traumatic brain injury. Failure on one or more of the freestanding and embedded measures of neuropsychological performance validity, reflecting inadequate motivation/effort, was an additional exclusion criterion. Performance validity was assessed using the Test of Memory Malingering (TOMM), the Reliable Digit Span test, and the California Verbal Learning Test—second edition Forced Choice Measure. Participants were excluded if any of these measures indicated suboptimal effort [27]. Participants were also excluded if glasses were necessary to clearly visualize calibration and visual stimulus targets during eye tracking. 

Data were available for sixteen participants; two were subsequently excluded due to poor-quality eye-tracking data. For the remaining 14 (median age 34 years, range 24–61; 6 women), concussion history consisted of either a single concussion (8 participants) or multiple concussion events (6 participants). The interval of time between the most recent concussion, eye tracking, and neuropsychological assessment was variable, ranging from 2 weeks to 84 months (mean time interval between concussion and neuropsychological assessment: 10.2 months, mean time interval between concussion and eye tracking: 11.7 months) (Table 1). All participants had self-reported ongoing symptoms related to their concussion at the time of assessment. 

### 2.2. Materials and Procedures

#### 2.2.1. KD Test and Eye Tracking

All participants had previously performed a digitized version of the KD test while simultaneously undergoing binocular eye movement recordings with the EyeLink 1000 Plus, an infrared-based video-oculographic camera system (SR Research, Mississauga, ON, Canada). A forehead rest was utilized for maximum head stability while simultaneously allowing for mouth movements required for number naming. The EyeLink sampled eye position at 500 Hz with a precision of 0.1 degrees. Participants completed an Eyelink standardized 13-point spatial calibration and validation procedure prior to each testing session. The 13-point serial target presentation calibration, rather than the traditional 9-point calibration, was utilized to ensure calibration across the entire display monitor. KD numbers were presented exclusively within the calibration region. Eye position was recorded continuously during onscreen presentation of all KD cards.

The KD test consisted of three computer-generated KD test cards that maintained consistency (e.g., numbers presented, inter-number spacing) with the spiral-bound version of the KD test [21,28]. After presentation of an initial demonstration card, the three test cards of the KD test were serially presented on the computer monitor. Participants were instructed to name the numbers on each card as quickly as possible. The total test time in seconds needed to name all the numbers on the three test cards (excluding time between cards) was recorded. The total number of errors was also recorded. The methodology of digitization of the KD test with simultaneous eye tracking and data analysis has previously been published [29]. 

#### 2.2.2. Neuropsychological Assessment

Neuropsychological measures included standardized tests of performance validity, processing speed, attention and working memory, perceptual reasoning, executive functioning, and emotional functioning (Table 2). Testing was performed as part of our interdisciplinary concussion center clinical neuropsychological testing battery and followed standardized administration procedures. 

Processing speed was measured using the Stroop Color and Word Tests (SCWT) Word and Color Scores, as well as the Wechsler Adult Intelligence Scale 4th edition (WAIS-IV) Coding subtest [30,31]. Attention and working memory were assessed using the WAIS-IV Digit Span Forward (DSF), Backward (DSB), Sequencing (DSS), and Total (DST), in addition to the Trail Making Test Part A (TMTA). Perceptual reasoning was examined using the Wechsler Abbreviated Scale of Intelligence—2nd edition (WASI-II) Matrix Reasoning subtest. Executive functioning was evaluated using the SCWT Interference score and the Trail Making Test Part B (TMTB). Emotional functioning was assessed using the Beck Anxiety Inventory (BAI) and the Beck Depression Inventory-II (BDI-II). Standardized administration according to testing instructions was followed for all validated measures of cognitive and emotional functioning. 

### 2.3. Data Analyses

Eye movement data were analyzed offline using custom MATLAB software (MathWorks, version 2020B, Massachusetts, MA, USA). Saccades were identified via an adaptive thresholding mechanism, and velocities and accelerations were computed from position traces using a low-pass differentiator [32]. ISIs were extracted for further analysis. Spearman correlations (non-parametric) were performed using each of the cognitive measures against the ISI values as continuous variables for the WAIS subtests. Parametric testing with Pearson correlations was used for timing data. 

## 3. Results

### 3.1. KD Times and ISI Values 

KD test times were substantially prolonged in this cohort of participants with a history of concussion and persistent symptoms to 72.6 (±20.7) sec relative to previously published KD test times in healthy individuals with no history of concussion (51.24 (±9.7) sec [29]—53.4 (±14.04) sec) [26]. ISIs were measured for each participant as the median interval between all task-specific saccades across the three test cards due to the expected substantial positive skew in the distribution of these values for all participants. The median ISI for this cohort was 379.1 (±199.1) msec, which is longer than typical ISIs in healthy individuals in our lab of 235.5 (±119.1) msec [29] and 286.1 (±49.7) msec [26].

### 3.2. ISI and Neuropsychological Assessments

**Figure 1:**
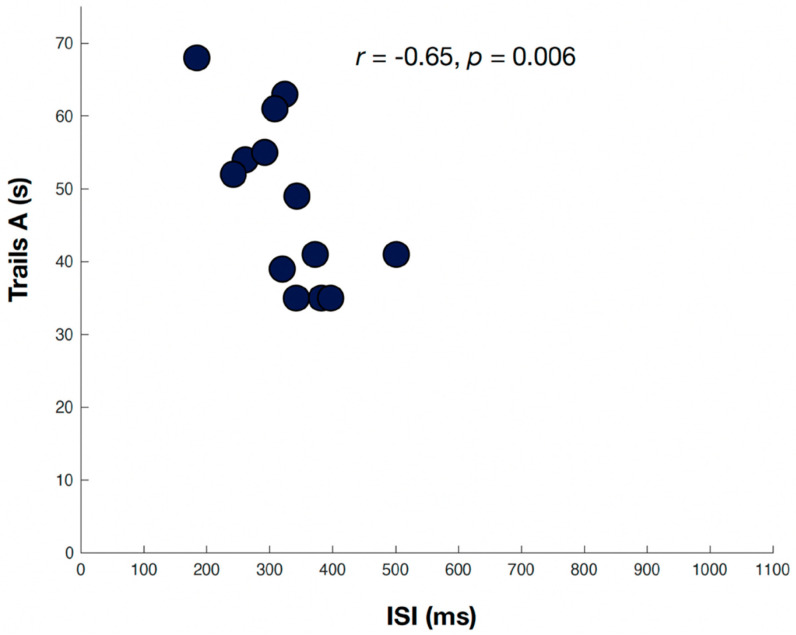
Relation of inter-saccadic intervals (msec) and the Trails Making Test Part A (T-score), a marker of attention/working memory. Two participants (grey circles) were excluded due to poor eye-tracking data quality.

**Figure 2:**
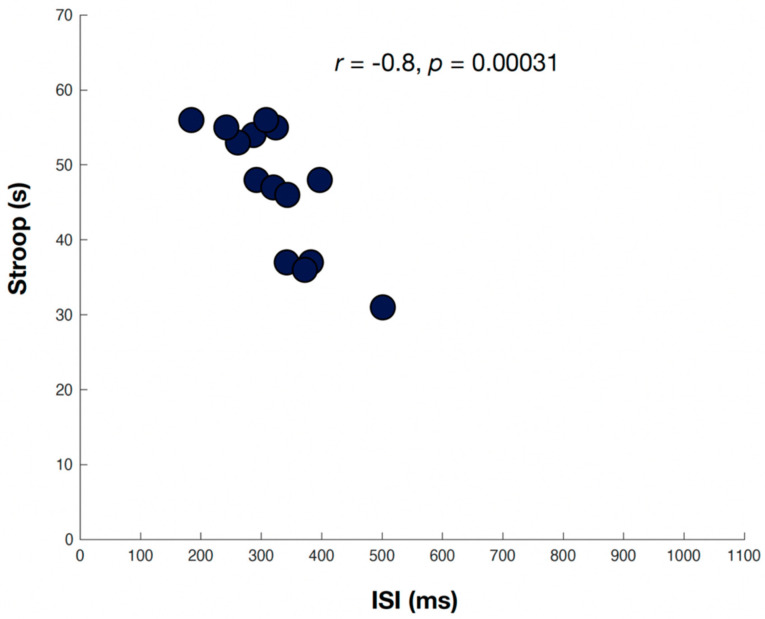
Relation of inter-saccadic intervals (msec) and the Stroop Color Word Interference Test (T-score), a marker of executive function. Two participants (grey circles) were excluded due to poor eye-tracking data quality.

Neuropsychological assessment scaled scores for assessments associated with greater ISI prolongation are shown in Table 3. Greater ISI prolongation was associated with lower scores in the cognitive domains of processing speed, attention/working memory, and executive function. The Spearman correlation coefficient was significant when comparing the ISI and TMTA (*r* = −0.65, *p* = 0.006) (Figure 1), DSF (*r* = 0.57, *p* = −0.017), DSB (*r*= −0.55, *p* = 0.021), and DST (*r* = −0.74, *p* = 0.001)—all tests of attention/working memory. Lower scores on SCWT Interference, a marker of executive function, were also significantly associated with ISI prolongation (*r* = −0.8, *p* = 0.0003) (Figure 2). The WAIS-IV Coding score, a marker of processing speed, was significantly associated with ISI prolongation (*r* = 0.72, *p* = 0.0017). The remaining measures showed a trend toward association, though they were not significant. 

## 4. Discussion

In this small pilot study, we sought to retrospectively explore the relationship between ISI prolongation during KD test performance and standardized neuropsychological assessments in a cohort of individuals with a history of concussion who had been evaluated in our concussion center. The aim was an initial exploration to advance understanding of the factors that may contribute to, and thus explain, slowed KD test performance following a concussion. Our data demonstrated that in this outpatient cohort with persistent symptoms, ISI prolongation during KD testing was associated with diminished neuropsychological function in the cognitive domains of processing speed, attention/working memory, and executive function. We will briefly review traditional applications of eye tracking and neuropsychological testing in concussion and then further consider their interactions and potential contributions to slowed KD test performance and ISI prolongation during KD testing in concussion. 

### 4.1. Eye Tracking and Neuropsychological Assessment in Concussion

There has been growing interest in, as well as debate about, visual symptoms, eye movements, and eye tracking applications in and after concussion over the past decade [33,34,35,36,37,38]. Given that saccadic eye movements are the eye movement type predominantly employed during the KD test, we limit the discussion here to eye-tracking studies of saccades. Brain networks that govern saccades are well-delineated, widely distributed, and extend from the frontal and parietal cortices down to the brainstem premotor nuclei. These nuclei ultimately initiate the motor command for a saccade in the ocular motor nuclei that send signals to the extraocular muscles [39]. 

Various subtypes of saccades can be assessed with eye tracking to probe the integrity of different regions of these saccadic networks. Most studies of saccades in concussions show that simple visually guided saccades are unaffected (i.e., not slowed), thus indicating that the immediate premotor structures in the brainstem that drive saccades are typically unaffected in acute concussion and chronic symptomatic states after concussion [40,41,42,43,44,45,46]. The exception to simple visually guided saccades being unaffected is the finding of increased saccadic latency (time between visual stimulus presentation and initiation of a saccade) for visually guided saccades in hyper-acute concussion, a finding which quickly resolves [47,48]. In keeping with studies largely showing normal visually guided saccades, saccade speeds have been shown to be normal during KD testing post-concussion, as well [26]. In contrast, abnormalities are often identified in attentionally-dependent saccade types that probe higher cortical, particularly prefrontal, structures involved in saccade generation, such as the frontal and supplementary eye fields and the dorsolateral prefrontal cortex (DLPC). These brain regions are particularly prone to traumatic brain injury [49]. Saccade types dependent on these higher cortical structures include memory-guided saccades (e.g., saccades to the remembered location of a previously present visual target) and antisaccades (e.g., saccades in the direction opposite to a suddenly appearing visual target); these saccade types assess cognitive functions such as short-term spatial memory, response inhibition, motor-sequence programming, visuospatial processing, and visual attention [42]. Increased saccadic latencies, more directional errors, and poorer spatial accuracy in these saccade types are an established indicator of suboptimal brain function in patients with acute concussion and chronic symptomatic states after concussion [40,42,50,51,52,53,54]. 

Neuropsychological testing is also widely utilized to identify suboptimal brain function in concussion and chronic symptomatic states after concussion and can assess cognitive, behavioral, and emotional aspects of functioning. Performance on neuropsychological assessments can be impacted by a range of variables, including mood, physical symptoms (e.g., headaches, fatigue, vestibular symptoms, etc.), education level, and premorbid conditions [55,56]. Processing speed and working memory are the most sensitive measures of cognitive dysfunction in concussion, though abnormalities in executive function, attention, and cognitive flexibility may be the most persistent cognitive deficits [30,56]. These deficits can affect performance on assessments such as the Stroop Color Word tests and the Trail Making Test Part A [54,57,58].

A few studies have assessed the relationship between eye movements and neuropsychological testing by either comparing the sensitivities of higher cortical saccade types with standard neuropsychological assessments for concussion diagnosis [42] or more directly considering higher cortical saccades as measures of neurocognitive dysfunction [54]. Abnormalities in memory-guided saccades and antisaccades have been shown to remain impaired longer and to correlate better with post-concussive symptoms and impaired activities of daily living than neuropsychological assessments in individuals with persistently symptomatic post-concussive states [42]. Abnormalities of antisaccades have been shown to correlate with greater symptom burden in acute concussion [54] and with poor performance on the Stroop test of executive function, which requires response inhibition. The dorsolateral prefrontal cortex (DLPFC), in particular, is known to play a key role in working memory and in inhibition of a reflexive saccade to the suddenly appearing visual target in the antisaccade task and may play a key role in deficits in concussion [59]. Indeed, the DLPFC has been shown to have transient alterations in its metabolic profile following head acceleration events, a proxy for sports-related concussion [60].

### 4.2. Interactions between KD Performance and Neuropsychological Assessments

The KD test and other rapid automatized naming tasks are performance measures that harness a number of different neurological systems, including vision and saccadic eye movements, cognitive aspects of attention and processing speed, and language function. As such, they have the capacity to capture dysfunction in concussion and have been shown to be sensitive measures for diagnosis on the sidelines of sport [16,21,22,23] and to be predictive of recovery in the outpatient arena [19]. To date, research on the exact contributing factors that underlie prolonged test times on these vision-based performance measures in the setting of concussion has been sparse.

Our focus has been on understanding eye-movement behaviors associated with slowed KD test times in concussion, which are predominantly correlated with longer ISIs [26]. Certainly, it is anticipated that neuropsychological abnormalities might be one of the factors contributing to prolonged test times. Indeed, it has been previously reported that longer (worse) KD completion times are associated with lower (worse) scores on the Sports Concussion Assessment Tool 2 (SCAT2), Standardized Assessment of Concussion (SAC) Immediate Memory Score, and on the overall SAC score [23,61]. In this exploratory pilot study, ISI prolongation during KD testing was associated with impaired neuropsychological function in the cognitive domains of processing speed, attention/working memory, and executive function. It is thus possible that the processing speed for the KD test may be adversely impacted by impaired visuospatial attention. KD performance requires a constant “updating” of attention and motor planning, and concussed individuals have been shown to have difficulty with visual disengagement [62]. We only included neuropsychological data interpreted as valid based on performance validity tests. Self-reported measures of mood were not significantly associated with ISI findings, suggesting interconnectivity of ISI and injury sustained by concussion that was independent of mood. Also notable in this study was the fact that the association between ISI and neuropsychological assessments was present even in the absence of objective cognitive impairment based on scoring parameters.

### 4.3. Study Limitations

Participants for this exploratory, retrospective pilot study were included based on the availability of relevant data for retrospective analysis, which led to a small number of participants being included and very high variability in the timing between the eye-tracking and neuropsychological assessments. Nonetheless, associations persisted in this pilot study and would likely be even more robust if timing intervals were standardized in future studies. In keeping with the retrospective nature of the study, the study population was also heterogeneous with regard to the number of concussions and time since the most recent injury. Thus, the generalizability of the results is presently unknown, and it was not possible to control for levels of fatigue or medications at the time of testing. Future prospective studies exploring the relationship between KD and neuropsychological testing performance will allow the opportunity for the inclusion of a control participant group and evaluation of the impact of age on performance. 

## 5. Conclusions

Quantitative assessment of the ISI during rapid automatized naming tests, likely in conjunction with other concussion-based diagnostics, is an objective, quantifiable eye-tracking metric of potentially high importance. This study provides preliminary evidence that cognitive dysfunction may be one element underlying prolonged ISI and KD test times after concussion. It is likely that other factors may also play a role. Given that all visual targets (e.g., all KD numbers) are displayed simultaneously during the KD test, we cannot directly measure true saccadic latency or assess capacity for disengagement from numbers. Exploration of these components will be our next step, as we assess the relationships between ISI and traditional measures of saccades, including the latencies of visually guided saccades and other saccade types, such as gap saccades (in which the fixation target disappears prior to the appearance of the visual target for saccade initiation, which facilitates disengagement from the prior number). A comprehensive understanding of the underlying components contributing to the prolongation of KD test times in and after concussion will help to elucidate what rapid number naming tasks are capturing and where these deficits may localize in the brain. 

## Figures and Tables

**Table 1:** Patient demographics and timing of assessments relative to last concussion.

Participant	Age (at Eye Movement Visit)	Sex *	Neuropsychological Testing (Relative to Most Recent Concussion)	Eye Movement Recording (Relative to Most Recent Concussion)
1	28	M	7 months	6 months
2	28	M	>24 months (2013—11/2015)	>24 months (2013—9/2015)
3	50	F	3 months	4 months
4	61	M	29 months	30 months
5	47	M	4 months	7 months
6	32	F	18 months	17 months
7	47	F	1 month	19 months
8	57	F	10 months	13 months
9	32	M	>24 months (2009—8/2016)	>24 months (2009—6/2016)
10	38	F	2 months	5 months
11	34	M	1 month	2 weeks
12	34	F	8 months	6 months
13	30	M	8 months	8 months
14	24	M	4 months	4 months

* M = male, F = female.

**Table 2:** Neuropsychological assessments utilized and the corresponding cognitive domain evaluated.

Cognitive Domain	Test *
Processing Speed	SCWT Word; SCWT Color; WAIS-IV Coding
Attention/Working Memory	WAIS-IV (Digit Span Forward; Digit Span Backward; Digit Span Sequencing; Digit Span Total); TMTA
Perceptual Reasoning	WASI-II Matrix Reasoning
Executive Functioning	SCWT Interference; TMTB
Emotional Functioning	BAI; BDI-II

* Abbreviations in Table: BAI = Beck Anxiety Inventory; BDI-II = Beck Depression Inventory, Second Edition; SCWT = Stroop Color and Word Test; TMTA = Trail Making Test A; TMTB = Trail Making Test B; WAIS = Wechsler Adult Intelligence Scale, Fourth Edition; WASI-II = Wechsler Abbreviated Scale of Intelligence, Second Edition.

**Table 3:** Neuropsychological assessment scaled or *T*-scores *.

Subject	Trail Making Test A (T-Score)	Digit Span Forward (SS)	Digit Span Backward (SS)	Digit Span Total (SS)	SCWT Interference (T-Score)	WAIS-IV Coding (SS)
1	76	9	8	9	51	15
2	68	11	14	12	60	10
3	35	10	13	11	51	6
4	35	5	7	5	42	5
5	54	10	14	12	46	15
6	63	12	10	10	56	9
7	55	11	8	12	50	12
8	41	11	8	9	50	8
9	52	12	14	13	67	16
10	35	8	10	8	57	11
11	61	12	12	14	52	11
12	41	8	10	9	59	6
13	39	12	12	12	54	10
14	49	9	10	11	49	9

* *T*-score from 20–30 or scaled score (SS) from 1 to 4 is between −3 standard deviation (SD) and −2SD. *T*-score from 31 to 40 or SS from 4 to 7 is between −2SD and −1SD. *T*-score from 41–50 or SS from 7 to 10 is between −1SD and 0SD. *T*-score from 51 to 60 or SS from 10 to 13 is between 0SD and +1SD. *T*-score from 61 to 70 or SS from 13 to 16 is between +1SD and +2SD. *T*-score from 71 to 80 or scaled score 16–19 is between +2SD and +3SD.

## Data Availability

Original data will be made available upon request.
